# The impact of S2 mutations on Omicron SARS-CoV-2 cell surface expression and fusogenicity

**DOI:** 10.1080/22221751.2023.2297553

**Published:** 2023-12-19

**Authors:** Alba Escalera, Manon Laporte, Sam Turner, Umut Karakus, Ana S. Gonzalez-Reiche, Adriana van de Guchte, Keith Farrugia, Zain Khalil, Harm van Bakel, Derek Smith, Adolfo García-Sastre, Teresa Aydillo

**Affiliations:** a Department of Microbiology, Icahn School of Medicine at Mount Sinai, New York, NY, USA; b Global Health and Emerging Pathogens Institute, Icahn School of Medicine at Mount Sinai, New York, NY, USA; c Graduate School of Biomedical Sciences, Icahn School of Medicine at Mount Sinai, New York, NY, USA; d Center for Pathogen Evolution, Department of Zoology, University of Cambridge, Cambridge, UK; e Department of Genetics and Genomic Sciences, Icahn School of Medicine at Mount Sinai, New York, NY, USA; f Icahn Genomics Institute, Icahn School of Medicine at Mount Sinai, New York, NY, USA; g Department of Medicine, Division of Infectious Diseases, Icahn School of Medicine at Mount Sinai, New York, NY, USA; h Department of Pathology, Molecular and Cell-Based Medicine, Icahn School of Medicine at Mount Sinai, New York, NY, USA; i The Tisch Cancer Institute, Icahn School of Medicine at Mount Sinai, New York, NY, USA

**Keywords:** SARS-CoV-2, Omicron, spike, cell–cell fusion, spike cell surface expression, “first-generation” Omicron sublineages, “second-generation” Omicron sublineages

## Abstract

SARS-CoV-2 Omicron subvariants are still emerging and spreading worldwide. These variants contain a high number of polymorphisms in the spike (S) glycoprotein that could potentially impact their pathogenicity and transmission. We have previously shown that the S:655Y and P681H mutations enhance S protein cleavage and syncytia formation. Interestingly, these polymorphisms are present in Omicron S protein. Here, we characterized the cleavage efficiency and fusogenicity of the S protein of different Omicron sublineages. Our results showed that Omicron BA.1 subvariant is efficiently cleaved but it is poorly fusogenic compared to previous SARS-CoV-2 strains. To understand the basis of this phenotype, we generated chimeric S protein using combinations of the S1 and S2 domains from WA1, Delta and Omicron BA.1 variants. We found that the S2 domain of Omicron BA.1 hindered efficient cell–cell fusion. Interestingly, this domain only contains six unique polymorphisms never detected before in ancestral SARS-CoV-2 variants. WA1^614G^ S proteins containing the six individuals S2 Omicron mutations were assessed for their fusogenicity and S surface expression after transfection in cells. Results showed that the S:N856K and N969K substitutions decreased syncytia formation and impacted S protein cell surface levels. However, we observed that “first-generation” Omicron sublineages that emerged subsequently, had convergently evolved to an enhanced fusogenic activity and S expression on the surface of infected cells while “second-generation” Omicron variants have highly diverged and showed lineage-specific fusogenic properties. Importantly, our findings could have potential implications in the improvement and redesign of COVID-19 vaccines.

## Introduction

The Omicron variant was first identified in South Africa and Botswana and classified as the fifth variant of concern (VOC) by the World Health Organization (WHO) on 26 November 2021 [1]. This novel variant, also known as B.1.1.529, rapidly spread worldwide and outcompeted previous circulating SARS-CoV-2 viruses. Since then, Omicron variant has evolved into different sublineages including the “first-generation” Omicron BA.1, BA.2, BA.4, BA.5 subvariants; and the recently emerged “second-generation” Omicron subvariants such as BQ., XBB. and EG. All these Omicron sublineages are characterized by an enhanced transmissibility, viral infectivity and increased resistance from neutralizing antibodies induced by previous infection and vaccination [2–5]. Some of these distinct virological features have been linked to the unusually high number of mutations contained in the spike (S) protein of Omicron compared to ancestor variants. The S glycoprotein is expressed as a homotrimer on the surface of the viral particle and therefore, is the major determinant of cell entry and tissue tropism. Each S monomer is heavily glycosylated, and it is composed of an antigenically variable S1 subunit and a more conserved S2 domain. Importantly, glycans have been shown to play a critical role in stabilizing the S structure as well as shielding the S to decrease antibody recognition [6,7]. Similar to other viral entry glycoproteins such as the influenza virus haemagglutinin protein [8,9], the S undergoes a cleavage activation process to infect the host cell that is driven by cellular proteases. The first cleavage occurs during biosynthesis and translocation of the S from the endoplasmic reticulum to the Golgi of the infected cell [10,11]. Here, furin proteases will cleave at the furin cleavage site present at the S1/S2 junction [12–14]. This first cleavage will prime the S protein for a subsequent cleavage at the S2’ site during viral entry. SARS-CoV-2 viruses can enter the cells through two different mechanisms: upon the interaction of the primed S with the angiotensin-converting enzyme 2 (ACE2) receptor at the host cell surface, the S2’ site will be exposed and cleaved by TMPRSS2 protease [12,15] at the cell surface; or endocytosis and subsequent interaction with cathepsin L in the endosome [16]. This second cleavage will trigger the fusion between the viral and cellular membranes. Upon infection, the S protein will be expressed on the surface of infected cells where it interacts with ACE2 receptor on the neighbouring cells and mediates cell–cell fusion [11,17,18]. A suboptimal coat protein I (COPI) recognition motif in the cytoplasmic tail of S has been suggested to be responsible for the S localization to the cellular plasma membrane [19]. Importantly, efficient proteolytic cleavage of the S protein by TMPRSS2 protease is required to induce syncytia formation, which has been associated with increased pathogenesis and disease severity [20,21]. However, the molecular mechanism regulating syncytia formation as well as the biological implications of this cell–cell fusion during SARS-CoV-2 infection are still not fully understood. We have previously demonstrated that polymorphisms in the S protein can strongly influence S cleavage efficiency and syncytia formation [22]. We found that the S:655Y mutation, a representative polymorphism of the Gamma lineage, enhanced viral replication, S protein cleavage and cell–cell fusion in a human airway epithelial system. Moreover, viral competition experiments in hamsters demonstrated that the S:655Y was transmitted more efficiently than the ancestor S:655H. Similar to other highly prevalent substitutions such as P681H/R and A701V, the S:655Y polymorphism is spatially located in close proximity to the furin cleavage site. We also previously found that all VOC tested in our study (Alpha, Beta, Gamma, Kappa and Delta) independently evolved to a convergent phenotype associated with an increased S cleavage and syncytia formation. Interestingly, both 655Y and P681H were later found to be present in the S protein of all Omicron subvariants. Additionally, and compared to the ancestral Wuhan-1 strain, all Omicron S proteins contain more than 30 mutations including amino acid substitutions, deletions, and insertions. Most of these polymorphisms are found in the S1 domain and most of them are shared among Omicron sublineages. This is the case for the K417N, N440K, T478K, E484A and N501Y mutations which have been previously associated with an enhanced ACE2 binding and/or immune evasion [23–25]. In contrast, other polymorphisms such as L452R and F486V are only present in Omicron BA.4 and BA.5 sublineages. The L452R mutation has been shown to enhance SARS-CoV-2 fusogenicity and infectivity while F486V confers escape from neutralizing antibodies [26–28]. Additional polymorphisms at S position 486 (F686S/P) can be found in XBB and EG subvariants and have been also associated to decrease antibody recognition [29]. On the other hand, only few amino acid changes can be found in the S2 domain, suggesting a more restricted mutational profile. The N764K, D796Y, Q954H and N969K are common in all “first-generation” and “second-generation” Omicron sublineages whereas N856K and L981F are exclusively found in the S2 domain of Omicron BA.1. Some preliminary studies have suggested that the S:N969K might mediate cell entry through the endosomal pathway [30]. Additionally, the T883I and I1210T polymorphisms have also been reported in the S2 domain of some XBB sublineages. However, the exact functional role of mutations in the S2 domain has not been elucidated yet.

Here, we characterized *in vitro* the S cleavage efficiency and fusogenic activity of different “first-generation” and “second-generation” Omicron sublineages. We showed that Omicron BA.1 subvariant had a decreased fusogenic activity despite containing the S:655Y and 681H polymorphisms. To investigate this attenuated fusogenic phenotype, we generated chimeric S proteins using combinations of WA1, Delta and Omicron BA.1 S1 and S2 domains. Results showed that the S2 domain of Omicron BA.1 decreased syncytia formation and reduced the S protein levels on the surface of transfected cells. Interestingly, two mutations located in the S2 domain, S:N856K and N969K, were responsible for this attenuated cell–cell fusion phenotype. Finally, we characterized different Omicron sublineages in the context of infection and observed that “first-generation” Omicron subvariants progressively evolved to an enhanced fusogenic activity and S cell surface expression while “second-generation” Omicron sublineages have highly diverged and showed lineage-specific fusogenic properties. Taken together, our results suggested that the S:N856K and N969K polymorphisms found in the S2 domain of Omicron BA.1 S protein significantly decreased the ability of the virus to induce syncytia formation. Importantly, these findings could have potential implications in the improvement and redesign of COVID-19 vaccines.

## Materials and methods

### Cell lines

VeroE6 cell line constitutively expressing full-length human TMPRSS2 (Vero-TMPRSS2) was purchased from BPS Bioscience (78081). Human HEK293T cells were originally purchased from the American Type Culture Collection (ATCC). Human A549 cells constitutively expressing human ACE2 receptor (A549-ACE2) were kindly provided by Dr. Lisa Miorin [31]. All cell lines were maintained in Dulbecco’s modified Eagle’s medium (DMEM) with glucose, L-glutamine, and sodium pyruvate (Corning, 10-017-CV) supplemented with 10% fetal bovine serum (FBS, Peak Serum), non-essential amino acids (Corning, 25-025-CI), penicillin (100 UI/mL) and streptomycin (100 UI/mL) (Corning, 30-002-CI). Additionally, Vero-TMPRSS2 media was supplemented with puromycin (3 ug/mL) (InvivoGen, ant-pr-1). All cell lines were grown at 37°C in 5% CO_2_ and supplemented with normocin (100 ug/mL) (InvivoGen, ant-nr-1) to prevent mycoplasma infection.

### Plasmids

Viral RNA from SARS-CoV-2 viruses was extracted using E.Z.N.A Viral RNA kit (Omega Bio-Tek, R6874). Full-length spike was amplified using the OneTaq One-Step RT–PCR Kit (NEB, E5315S) and spike cDNA fragments were cloned into the pCAGGS expression vector using In-Fusion HD cloning kit (Takara, 638948). Mutations in the S sequence were introduced via PCR with overlapping primers. Spike chimeras were generated by amplifying S1 or S2 coding sequences and assembling them in different combinations using the NEBuilder HiFi DNA Assembly cloning kit (NEB, E5520S) in pCAGGS expression plasmids. All plasmids were sequence-confirmed (Psomagen) and data were analysed using SnapGene 5.3.2 software**.**

### Viruses

SARS-CoV-2 ancestral WA1^614G^, Delta, Omicron BA.1 (BA.1.17.2), BA.2 (BA.2.9.2), BA.4 (BA.4.1), XBB.1, XBB.1.5 (XBB.1.5.4) and EG.1 variants used in this study were originally isolated from nasopharyngeal swabs of COVID-19 infected individuals as previously described [32] and kindly provided by Dr. Viviana Simon. Specimens were collected as part of the routine SARS-CoV-2 surveillance conducted by the Mount Sinai Pathogen Surveillance programme (IRB approved, HS#13-00981). SARS-CoV-2 BA.5 (BA.5.2.1) subvariant was obtained from BEI resources. To generate viral stocks, Vero-TMPRSS2 cells were infected at a MOI of 0.01 and maintained in infection media (DMEM with glucose, L-glutamine, and sodium pyruvate supplemented with 2% FBS, non-essential amino acids, HEPES, penicillin (100 UI/mL) and streptomycin (100 UI/mL)) at 37°C in 5% CO_2_. Cells were monitored for cytopathic effect and viral supernatants were collected at day 3 post-infection for WA1^614G^ and Delta variants and at day 4–5 post-infection in the case of all Omicron subvariants. Viral supernatants were clarified of cell debris by spin down and aliquots were stored at −80°C. All viral stocks were sequence-confirmed by next-generation sequencing. Experiments in this study were performed in the biosafety level 3 (BSL-3) facility following Icahn School of Medicine biosafety guidelines.

### Plaque assay analysis

SARS-CoV-2 plaque assays were performed as previously described [22,32]. Briefly, 320.000 Vero-TMPRSS2 cells per well were seeded in a 12 well-plate and cultured at 37°C in 5% CO_2_ overnight. The following day, ten-fold serial dilutions of virus supernatant were performed in infection media and inoculated onto the Vero-TMPRSS2 cell monolayer. After 1 h incubation at 37°C, the inoculum was removed, and cell monolayers were overlaid with minimum essential media (MEM) containing 2% FBS and purified agar (OXOID) at a final concentration of 0.7%. After 3 days at 37°C, plates were fixed with 10% formaldehyde. Then the overlay was removed, and plaques were immunostained. Briefly, cells were blocked in 5% non-fat dry milk-containing Tris-buffered saline with 0.1% Tween-20 (TBST) for 1 h. Next, cells were incubated with anti-SARS-CoV-2 NP antibody (1C7C7, kindly provided by Dr. Moran) at a 1:1000 dilution in 1% milk-TBST for 1 h at RT. Then, cells were washed twice with PBS and stained with secondary IgG-HRP antibody (abcam, 6823) at a dilution of 1:5000 in 1% milk-TBST and incubated for 1 h at RT. Finally, cells were washed twice with PBS and plaques were visualized using TrueBlue substrate (KPL-Seracare, 5510-0030). Viral titres were calculated considering the number of cells per well and expressed as plaque-forming units (PFU)/mL.

### Infection of cell cultures

320.000 Vero-TMPRSS2 cells per well or 250.000 A549-ACE2 cells per well were seeded in a 12 well-plate and cultured at 37°C in 5% CO_2_ overnight. The following day, Vero-TMPRSS2 cells were infected at an MOI 0.01 and A549-ACE2 cells were infected at an MOI 0.1 with the different SARS-CoV-2 variants for 1 h. Then, cells were washed twice with PBS and maintained in infection media until samples were collected for Western Blot analysis or cells were fixed for immunofluorescence assay and visualization by confocal microscopy.

### Western blotting

Western Blot analysis was performed as previously described [22,32]. Briefly, viral supernatants and cell extracts from Vero-TMPRSS2 and A549-ACE2 infected cells were collected after 48- and 72-hours post-infection, respectively. Samples were lysed with RIPA buffer (Sigma, R0278) containing EDTA-free protease inhibitor cocktail (Roche, 04693132001) and 10% SDS (Invitrogen, AM9822) to a final concentration of 1%. Then, samples were centrifuged for 10 min at 4°C at maximum speed and boiled for 10 min at 100°C Lane Marker Reducing Sample Buffer (Thermo Fisher, 39000). Samples were run on SDS-PAGE electrophoresis using precast 10% TGX gels (BioRad, 4561036) and transferred to polyvinylidene fluoride (PVDF) membranes (BioRad, 704156) using BIO-RAD semi-dry transfer system. Then, membranes were fixed with 10% methanol for 1 min and blocked with 5% non-fat dry milk- TBST for 1 h in shaking and room temperature (RT). Next, membranes were incubated with primary antibodies overnight at 4°C followed by incubation with secondary antibodies in a 3% milk diluted in TBST for 1 h at RT. Primary antibodies against SARS-CoV-2 Spike S2 protein (ThermoFisher, MA5-35946 or SinoBiological, 40590-D001) and nucleocapsid (Novus Biologicals, NB100-56576) were purchased from the indicated suppliers and used at a dilution of 1:3000 and 1:2000, respectively. Antibodies against TMPRSS2 protease (Millipore, MABF2158) and ACE2 receptor (GeneTex, 101395) were utilized at a dilution 1:1000 and used as biotinylation surface controls. Anti-mouse secondary IgG-HRP antibody (Abcam, 6823) was used at a dilution 1:5000 to detect SARS-CoV-2 Spike protein and anti-rabbit secondary IgG-HRP antibody (Kindle Biosciences, R1006) at 1:2000 to detect SARS-CoV-2 nucleocapsid. B-actin was detected using IgG-HRP antibody (Cell Signalling; 5125) at a dilution of 1:1000.

### Immunofluorescence

320.000 Vero-TMPRSS2 cells per well or 250.000 A549-ACE2 cells per well were seeded in a 12 well-plate glass-bottom plate and infected as described above. After 24 h post-infection, Vero-TMPRSS2 cells were fixed with 10% formaldehyde and permeabilized with 0.1% Triton X-100 for 10 min. Alternatively, A549-ACE2 cells were fixed and permeabilized after 48 h post-infection. Next, cells were washed twice with PBS and incubated with primary antibodies against S2 spike (SinoBiological, 40590-D001) and nucleoprotein polyclonal anti-serum diluted in 1% bovine serum albumin (BSA) for 1 h at RT. Then, cells were washed and stained with secondary antibodies anti-mouse Alexa Fluor-488 (ThermoFisher, A11013) and anti-Rabbit Alexa Fluor 568 (ThermoFisher, A11011) in 1% BSA for 1 h at RT. DAPI (4¢,6-dia-midino-2-phenylindole) was used to visualize the nucleus.

### Cell surface biotinylation assay

To evaluate the S expression on the surface of infected cells, a modified version of the Pierce™ Cell Surface Biotinylation and Isolation Kit (Thermo-Fisher, A44390) was used. Briefly, 1 million Vero-TMRSS2 cells per well or 650.000 A549-ACE2 cells per well were seeded in a 6 well-plate and cultured at 37°C in 5% CO_2_ overnight. The following day, cells were infected at an MOI 1 with the different SARS-CoV-2 variants. After 1 h incubation, cells were washed and maintained in infection media. Media was removed from Vero-TMPRSS2 cells and A549-ACE2 cells after 24- or 48-hours post-infection, respectively and, cells were washed with PBS. Then, Sulfo-NHS-SS-Biotin diluted in PBS was added to the wells and incubated for 10 mins at RT to label surface proteins. Next, labelling solution was removed, and cells were washed twice with cold TBS. Cells were scraped and lysed with RIPA buffer containing EDTA-free protease inhibitor cocktail and labelled proteins were isolated with NeutrAvidin Agarose beads. Finally, reducing agent Dithithreitol (DTT) was used to elute surface proteins for subsequent Western Blot analysis.

### Generation of GFP-split stable cell lines

A human codon-optimized GFP1-10 GeneArt DNA fragment with N-terminal HA-tag was synthesized (ThermoFisher) and cloned into XhoI/NotI site of pLVX-IRES-Neo plasmid (Takara, 632181) using the NEBuilder HiFi DNA Assembly Cloning Kit (NEB, E5520S). BSR-GFP11-flag was subcloned from pQCXIP-BSR-GFP11 (Addgene, 68716) plasmid into the XhoI/NotI site of pLVX-IRES-Neo plasmid. For lentiviral production, 6 million HEK293T cells were co- transfected in suspension with 3 μg lenti-gag/pol, 3 μg pMD2-VSV-G and 5 μg pLVX-IRES-Neo vector containing the GFP split constructs. Lentivirus particles were harvested at 48 and 72 h post-transfection and filtered using a 0.45 μm filter (Whatman). Vero-TMPRSS2 cells were then transduced with lentivirus in the presence of 8 μg/ml polybrene. Vero-TMPRSS2-HA-GFP1-10 cells were selected and maintained in DMEM supplemented with 10% FBS, 1% NEAA, 1% L-glutamine, 1% HEPES and containing 3 μg/ml puromycin and 500 μg/ml geneticin (ThermoFisher). Vero-TMPRSS2-BSR-GFP11-flag cells were selected and maintained in DMEM supplemented with 10% FBS, 1% NEAA, 1% L-glutamine, 1% HEPES and containing 3 μg/ml puromycin and 10 μg/ml Blasticidin (ThermoFisher).

### Cell–cell fusion assays

30,000 Vero-TMPRSS2 cells (15,000 VeroE6-TMPRSS2-HA-GFP1-10 and 15,000 VeroE6-TMPRSS2-BSR-GFP11-flag) were reverse transfected in a 96 well-plate (Greiner, 655090) with 100 ng pCAGGS- spike using TransIT-LT1 (Takara; MIR2300). After 24 h, cells were fixed with 4% paraformaldehyde in PBS for 10 min and permeabilized with 0.1% Triton X-100 for 10 min. DAPI was used to visualize the nucleus. GFP signals were quantified as a proxy of syncytia formation using the Celigo Image Cytometer (Nexcelom).

### Flow cytometry (FACS) for cell surface staining

HEK293T cells were transfected in 12 well-plates with 500 ng pCAGGS-Spike using Trans-LT1 (Mirus). 24 h post-transfection, cells were washed once with PBS and detached with 0.25% trypsin-EDTA (ThermoFisher). Trypsin was then inactivated using DMEM supplemented with 10% FBS. Cells were washed two more times by resuspension with Cell Staining Buffer (Biolegend, 420201). Next, cells were centrifuged at 1200 rpm for 5 min and stained with anti-SARS-CoV-2 S antibody (SinoBiological, 40590-D001) diluted in Cell Staining Buffer for 1 h at 4°C. After two washes with Cell Staining Buffer, cells were incubated with Goat anti-Human IgG (H + L) Cross-Adsorbed Secondary Antibody, Alexa Fluor™ 488 (ThermoFisher, A-11013) diluted in Cell Staining Buffer for 1 h at 4°C. Dead cells were excluded from the analysis using LIVE/DEAD™ Fixable Near-IR Dead Cell Stain Kit (ThermoFisher). After three final washes with Cell Staining Buffer, surface SARS-CoV-2 spike levels were analysed using a Beckman Coulter Gallios Flow Cytometer. Typically, 5000–10,000 live cells were acquired, and SARS-CoV-2 spike levels were analysed using FlowJo v10 Software.

### Illumina sequencing

Viral RNA from SARS-CoV-2 stocks was extracted using Omega E.Z.N.A Viral RNA kit (R6874) following manufacturer’s instructions. Samples for sequencing were prepared using whole-genome amplification with custom-designed tiling primers [33] and the Artic Consortium protocol (https://artic.network/ncov-2019), with modifications. Briefly, cDNA synthesis was performed with random hexamers and ProtoScript II (New England Biolabs, cat. E6560) using 7 μL of RNA according to manufacturer’s recommendations. The RT reaction was incubated for 30 min at 48°C, followed by enzyme inactivation at 85°C for 5 min. Targeted amplification was performed as previously described [34] with modifications to prevent amplicon dropout for the Omicron BA.* lineages [35]. Next, amplicons were visualized on a 2% agarose gel and cleaned with Ampure XT beads. Amplicon libraries were prepared using the Nextera XT DNA Sample Preparation kit (Illumina, cat. FC-131-1096), as recommended by the manufacturer and sequenced on an Illumina MiSeq (Paired-end, 2 × 150 bp). Finally, to assembly SARS-CoV-2 genomes a custom reference-based analysis pipeline was used [35].

### Phylogenetic ranking of mutations

Evolutionary convergence was calculated by reconstructing ancestral states at internal nodes of the SARS-CoV-2 phylogenetic tree by maximum parsimony and counting the number of distinct times a substitution occurred. Substitutions caused by CT and GT nucleotide substitutions have higher levels of convergent evolution on average due to the elevated rates of CT and GT nucleotide substitutions relative to other nucleotide substitutions. This was considered when interpreting convergence and frequency data to produce the selection of substitutions.

### Statistical analysis

Western blot quantification was performed using ImageJ and the level of full length and cleaved spike protein were normalized to nucleocapsid protein. Statistical analysis was performed using GraphPad Prism 9. One-way ANOVA or two-tailed t student was performed to compare mean differences in fusogenicity between S mutants and WT S proteins. Statistical significance was considered when *p* ≤ 0.05 (*p < 0.05, **p < 0.01, ***p < 0.001, ****p < 0.0001, ns, not significant).

## Results

### Fusogenic activity of single point mutations around the furin cleavage site of the S protein

**Figure 1.**
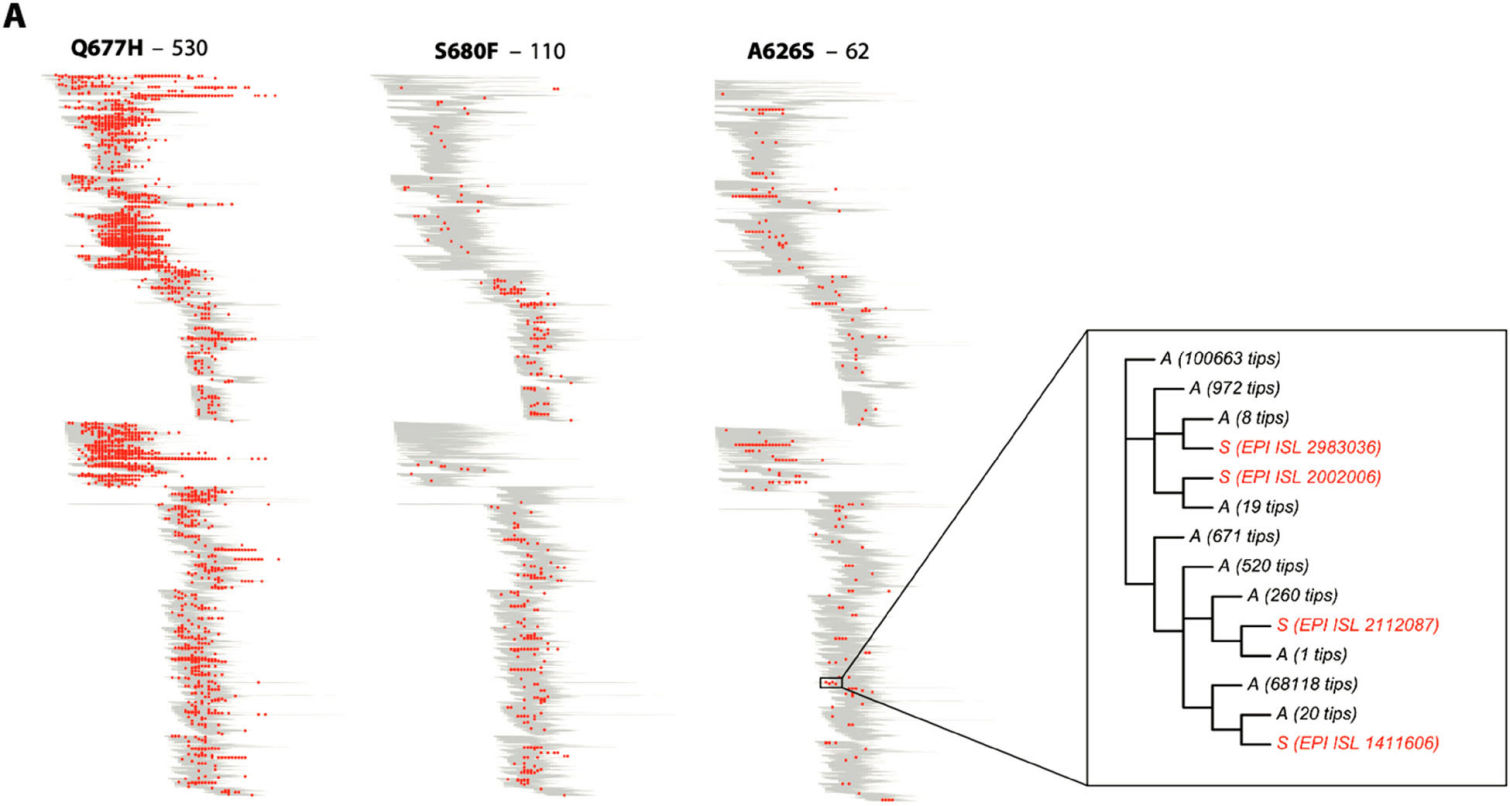
Phylogenetic trees showing the distribution of SARS-CoV-2 sequences containing the Q677H, S680F, and A626S spike substitutions. (A) Trees were generated using a total of 2,641,451 viral sequences downloaded from GISAID database on 3 September 2021. The Q677H, S680F, and A626S mutations were selected as representative S polymorphisms with high, medium, and low number of evolutionary occurrences, respectively. Above each tree, the estimated number of independent evolutionary occurrences of the substitution is given. Viral sequences containing the corresponding S polymorphisms are shown in red dots. An exploded view of part of the phylogenetic tree is shown, showing 4 independent occurrences of the A626S substitution.

**Figure 2.**
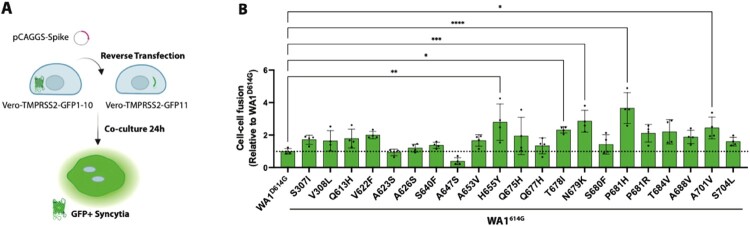
Fusogenic activity of single point mutations around the furin cleavage site of the S protein. (A) Schematic representation of the cell-cell fusion assay. (B) Quantification of syncytia formation of WA1^614G^ S and WA1^614G^ S containing single amino acid substitutions around the furin cleavage site. Cell-cell fusion was represented as GFP positive area and values normalized to WA1^614G^ S. Shown are the means and standard deviation (SD) of four replicates. One-way ANOVA with Dunnett’s multiple comparison test was performed to compare mean differences between each S mutant and WA1^614G^ S. Statistical significance was considered when *p* ≤ 0.05 (*p < 0.05, **p < 0.01, ***p < 0.001, ****p < 0.0001).

We previously showed that the furin cleavage site of SARS-CoV-2 viruses plays a crucial role in S protein cleavage and syncytia formation [13,14,22]. Importantly, an intact furin site is also required for viral transmission *in vivo* [36]. It is critical to monitor and characterize the potential impact of emerging S mutations close to the furin cleavage site on emerging SARS-CoV-2 variants. We performed a comprehensive phylogenetic analysis to identify emerging S mutations in close proximity to the furin cleavage site. For this, we used 2,641,451 available viral sequences from the GISAID database until September 2021. We then selected a subset of 21 S mutations for *in vitro* characterization by a quantitative split-GFP cell–cell fusion assay (Table 1). Mutations were selected based on the frequency of the substitution, its position in the secondary structure and the evolutionary convergence of the mutation as a metric to estimate the independent occurrence of a given polymorphism (Figure 1(A)). Next, we introduced each candidate point mutation into the WA1 (USA-WA1/2020) S protein harbouring the D614G mutation (WA1^614G^). We used the WA1^614G^ backbone since the S:614G mutation is a fixed polymorphism present in all current SARS-CoV-2 circulating variants that became dominant early during the COVID-19 pandemic. Spike proteins containing the candidate mutations were cloned into the pCAGGS expression vector and a Vero-TMPRSS2 GFP complementation assay was used to quantify syncytia formation (Figure 2(A)). Figure 2(B) shows GFP expression as a proxy of syncytia formation for each of the S protein tested and normalized to WA1^614G^ S. Results showed that 17 out of the 21 proposed mutations increased cell–cell fusion when introduced into WA1^614G^ S protein. Consistent with our previous findings [22], S proteins harbouring H655Y, P681H and A701V mutations were around 3-4-fold more fusogenic compared to WA1^614G^ S protein. Additionally, we found that the T678I and N679K substitutions were strong enhancers of syncytia formation with GFP expression levels similar to H655Y polymorphism. Interestingly, H655Y, N679K, P681H mutations are present in S protein of all circulating Omicron BA.1 variants, while the A701V substitution can be found in only few Omicron BA.1 sublineages. To investigate the epistatic effect of mutations around the furin cleavage site, we next generated a WA1^614G^ S protein harbouring the highly fusogenic H655Y, N679K, P681H mutations, and assessed its ability to induce syncytia formation (Supplementary Figure 1A-B). We found no statistical differences between the triple WA1^614G^- H655Y, N679K, P681H S mutant and the WA1^614G^ S proteins containing each individual mutation. However, we observed that the fusogenic activity of an Omicron BA.1 S protein containing the ancestral amino acids present in WA1 S protein at these three positions (BA.1-Y655H-K679N-H681P) was significantly decreased as compared to Omicron BA.1 wild type S protein (Supplementary Figure 1C), supporting the role of these S mutations in enhancing cell–cell fusion. Moreover, our results showed that S:A623S, A626S and S640F mutations had a minimum impact on the fusogenicity of WA1^614G^ S whereas the A647S polymorphism, a buried residue in the S protein, drastically decreased cell–cell fusion (Figure 2(B)).

**Table 1.** Ranking of high prevalent mutations around the furin cleavage site of the S protein.

Substitution	Frequency of substitution	Independent occurrences	Function
Q677H	34494	530	Increases viral infectivity and syncytium formation [54]
Q675H	20040	343	Improves spike interaction with the furin binding pocket [55]
P681H	1061155	288	Enhances S protein cleavage and syncytia formation [22,56]
P681R	540371	156	Enhances viral fitness and S protein cleavage [38,57]
N679K	2990	68	Increases S protein cleavage [58]
T678I	1876	126	Unknown
A684V	1683	132	Possible interaction with P681R to help furin binding [59]
A688V	10214	322	Unknown
S680F	1322	116	Unknown
Q613H	5200	359	Located next to D614G
S640F	4596	287	Unknown
H655Y	71206	268	Increases S cleavage, syncytia formation and viral transmission [22]
A701V	60499	231	Enhances syncytia formation [22]
A653V	5398	215	Buried residue, side chain interacts with furin loop. Unknown function
V622F	5598	209	Unknown
S704L	3445	199	Unknown
V308L	3077	246	Unknown
T307I	2232	134	Unknown
A647S	1132	153	Buried residue. Unknown function
A623S	1026	119	Unresolved in Cryo-EM, unknown function
A626S	1831	62	Decrease binding efficiency of vaccinated sera [60]. Unresolved in Cryo-EM

### The Omicron BA.1 subvariant shows attenuated fusogenicity in vitro compared to SARS-CoV-2 ancestral strains

**Figure 3.**
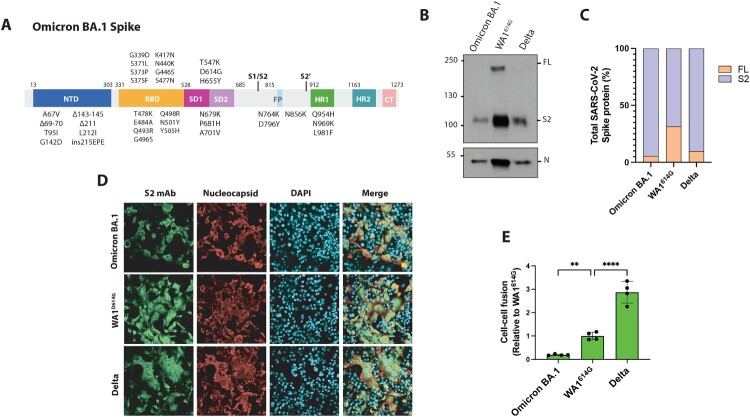
Omicron BA.1 subvariant is efficiently cleaved but shows attenuated fusogenicity. (A) Mutations present in Omicron BA.1 S protein. (B) Western Blotting of S protein cleavage of Omicron BA.1, Delta and ancestral WA1^614G^ variant. Infections were performed in Vero-TMPRSS2 cells at an MOI of 0.01 and supernatants were collected at 48 h.p.i. Full-length (FL) spike protein (180 kDa), S2 cleaved spike (95 kDa), and nucleocapsid (N; 50 kDa) were detected using specific antibodies. Levels of N protein were used as loading control. (C) Quantification of full-length and S2 cleaved spike protein. Spike protein levels were normalized to nucleocapsid expression. (D) Syncytia formation induced by Omicron BA.1, Delta and WA1^614G^ variants. Vero-TMPRSS2 cells were infected at an MOI of 0.01 and after 24 h.p.i, cells were fixed, and immunofluorescence assay was performed. Spike protein was detected using a S2 monoclonal antibody (green), N protein was detected using a polyclonal antiserum (red), and DAPI was used to stain the nucleus. Images were obtained using confocal microscopy. Scale bars, 50 μm. (E) Quantification of syncytia formation by each S variant represented as GFP expression normalized to WA1^614G^ S. Shown are the means and SDs of four replicates. One-way ANOVA with Dunnett’s multiple comparison test was performed to compare mean differences between each S variant and WA1^614G^ S. Statistical significance was considered when *p* ≤ 0.05 (*p < 0.05, **p < 0.01, ***p < 0.001, ****p < 0.0001).

A total of 30 amino acid substitutions, 6 amino acid deletions and 3 amino acid insertions can be found in Omicron BA.1 S compared to ancestral strains (Figure 3(A)). Interestingly, this variant contains the S:655Y, P681H and A701V polymorphisms which are known for their role on enhancement of S protein cleavage and syncytia formation [22,37,38]. Additionally, the N679K mutation is also present, suggesting that the S protein of Omicron BA.1 would be efficiently cleaved and highly fusogenic (Figure 2(B)). We performed infections in Vero-TMPRSS2 cells at an MOI of 0.01 with Omicron BA.1 variant and included the ancestral WA1^614G^ and Delta VOC as controls. After 48 h post-infection (h.p.i), supernatants from infected cells were collected and subjected to Western Blot analysis to assess the cleavage ability. As shown in Figure 3(B), Omicron BA.1 was efficiently cleaved and a band corresponding to the S2 cleaved form of the S protein (100 KDa) was detected. Similarly, we observed the S2 cleaved form in WA1^614G^ and Delta controls. Consistently, quantification of full-length and cleaved S protein showed that more than 90% of the Omicron S detected was in its cleaved form, indicating that Omicron S can get efficiently cleaved by cellular proteases (Figure 3(C) and Supplementary Figure 2A-B). Next, we investigated the syncytia formation induced by these variants. First, we performed immunofluorescence staining in Vero-TMPRSS2 infected cells after 24 h.p.i. Unexpectedly, Omicron BA.1 was poorly fusogenic and induced lower levels of syncytia formation compared to WA1^614G^ and Delta controls (Figure 3(D)). Next, we quantified the cell–cell fusion induced by the S proteins of these variants using the split-GFP complementation assay. For this, we cloned and transfected Omicron BA.1, Delta and WA1^614G^ S-pCAGGS plasmids into Vero-TMPRSS2-GFP1-10 and Vero-TMPRSS2-GFP11 cell lines. After 24 h, we measured GFP signal from each S variant and differences in fusogenicity were then calculated relative to WA1^614G^ S. Consistent with our infection experiments, we observed that Omicron BA.1 S induced lower levels of cell–cell fusion compared to WA1^614G^ and the highly fusogenic Delta variant (Figure 3(E)). Overall, these results demonstrate that Omicron BA.1 is efficiently cleaved but has attenuated fusogenicity.

### Specific mutations located in the S2 domain of Omicron BA.1 subvariant reduce S cell surface expression and fusogenic activity

**Figure 4.**
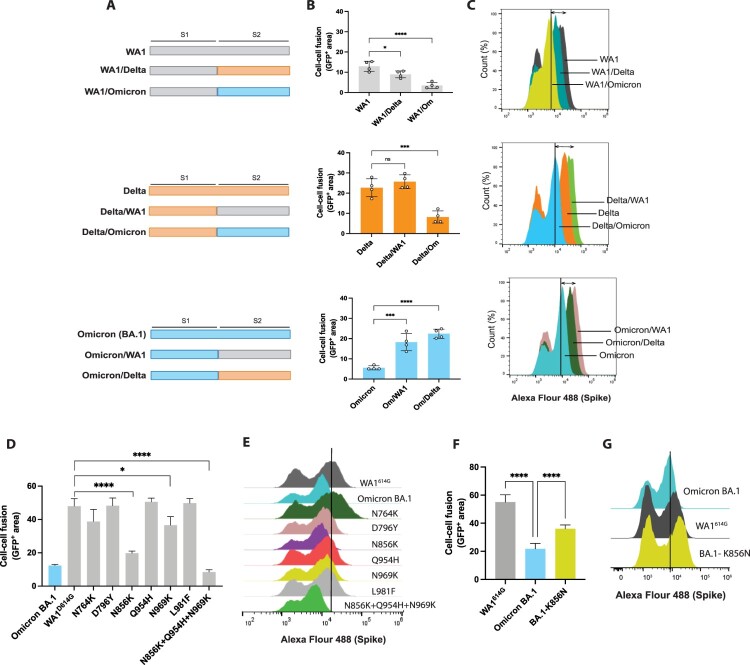
Specific mutations located in S2 domain of Omicron BA.1 variant reduce S cell surface expression. **(**A) Schematic representation of the S protein chimeras generated in this study. (B) Quantification of cell-cell fusion represented as GFP expression of each wild-type and chimeric S proteins. Shown are the means and SDs of four replicates. One-way ANOVA with Dunnett’s multiple comparison test was performed to compare mean differences between each chimeric S protein and corresponding wild type S variant. Statistical significance was considered when *p* ≤ 0.05 (*p < 0.05, **p < 0.01, ***p < 0.001, ****p < 0.0001). (C) Spike surface expression of chimeric and wild type proteins analysed by flow cytometry. HEK293T cells were transfected with the corresponding S proteins and after 24 h, cells were stained with anti-S antibody. (D) Quantification of syncytia formation of Omicron BA.1 S, WA1^614G^ S and WA1^614G^ S containing the different mutations found in Omicron BA.1 S2 domain. Cell-cell fusion was represented as GFP positive area. Shown are the means and SDs of three replicates. One-way ANOVA with Dunnett’s multiple comparison test was performed to compare mean differences between WA1^614G^ S and each WA1^614G^ S mutant. Statistical significance was considered when *p* ≤ 0.05 (*p < 0.05, **p < 0.01, ***p < 0.001, ****p < 0.0001). (E) Spike surface expression of S proteins analysed by flow cytometry. HEK293T cells were transfected with the corresponding S proteins and after 24 h, cells were stained with anti-S antibody. (F) Quantification of syncytia formation of WA1^614G^ S, Omicron BA.1 S and Omicron BA.1 S protein containing the ancestral amino acid present in WA1 S protein at position 856 (S: K856N). One-way ANOVA with Dunnett’s multiple comparison test was performed to compare mean differences between Omicron BA.1 S and WA1^614G^ or Omicron BA.1- K856N S mutant. Statistical significance was considered when *p* ≤ 0.05 (*p < 0.05, **p < 0.01, ***p < 0.001, ****p < 0.0001). (G) Spike surface expression of Omicron BA.1, WA1^614G^ and Omicron BA.1-K856N S proteins analysed by flow cytometry. HEK293T cells were transfected with the corresponding S proteins and after 24 h, cells were stained with anti-S antibody.

Most of the mutations present in Omicron BA.1 S protein are found in the S1 domain, especially in the receptor-binding domain (RBD). These mutations are critical for both receptor binding and viral escape to neutralizing antibodies [2,3,39,40]. In contrast, the S2 subunit, which is responsible for viral fusion with the host cell membrane, contains only 6 unique mutations: N764 K, D796Y, N856K, Q954H, N969K, and L981F. Interestingly, these mutations were not previously detected in other preceding SARS-CoV-2 variants. Although Omicron BA.1 harbours S: H655Y, N679K, P681H and A701V mutations, our data shown in Figure 3(D–E) demonstrated that this variant induced lower levels of syncytia formation. We next investigated whether the S2 domain could drive this observed phenotype. For this, we generated S protein chimeras using combinations of the S1 and S2 domains from WA1, Delta and Omicron BA.1 variants (Figure 4(A)). The fusogenic activity of these chimeric S proteins was compared to the wild-type S proteins in our cell–cell fusion assay (Figure 4(B)). Swapping the S2 domain of WA1 for Omicron BA.1 S2 domain drastically decreased the S-mediated cell–cell fusion. Similar results were found for Delta, and substitution of the S2 domain of Delta for Omicron BA.1 remarkably reduced the fusogenic activity. Conversely, we observed that chimeric proteins harbouring Omicron BA.1 S1 domain and the S2 domain of WA1 or Delta significantly increased their ability to induce syncytia compared to Omicron wild type demonstrating that the S2 domain of Omicron BA.1 is responsible for the attenuated fusogenicity of this variant. Syncytia formation is mediated by the interaction between the S protein expressed on the cell surface of infected cells and the ACE2 receptor on neighbouring cells. Since our results have indicated that the S2 domain of Omicron BA.1 subvariant decreases cell–cell fusion, we next assessed whether this was caused by lower expression levels of Omicron S protein on the cell surface. To test this, we transfected HEK293T cells with our chimeric and wild-type S proteins and stained the cells using specific anti-S2 antibody to quantify S protein surface expression by flow cytometry (Figure 4(C) and Supplementary Table 1). In the case of WA1 S, we observed that S protein levels on the surface of transfected cells were decreased when WA1 S2 domain was swapped for Delta or Omicron S2 subunit. Similarly, we detected lower amounts of S on the cell surface when Delta S2 was swapped for Omicron S2 domain. However, chimeric Delta S harbouring WA1 S2 showed slightly higher levels of S surface expression compared to Delta wild type. Interestingly, swapping the S2 domain of Omicron BA.1 for Delta or WA1 S2 subunit increased the S expression on the surface of transfected cells. Taken together, our results suggest that the S2 domain of Omicron BA.1 S hinders efficient cell–cell fusion by decreasing the S protein expression on the cell surface.

Next, we assessed the contribution of specific mutations located in the S2 domain of Omicron BA.1 to cell–cell fusion and S surface expression. For this, we generated S plasmids containing the 6 individual S2 point mutations (N764K, D796Y are N856 K, Q954H, N969 K, and L981F) into the WA1^614G^ S backbone and quantified their ability to induce syncytia in our cell–cell fusion assay (Figure 4(D)). Omicron BA.1 S and WA1^614G^ S were also included as controls. Interestingly, we observed that the N856K and N969K mutations significantly decreased the fusogenic activity of WA1^614G^ S. Additionally, a combination of the N856K, Q954H and N969 K polymorphisms in the WA1 backbone showed a bigger impact and drastically reduced S fusogenicity to the same levels as wild type Omicron BA.1 S. We then assessed whether these mutations may influence the levels of S protein expressed on the surface of transfected cells (Figure 4(E) and Supplementary Table 1). Our results showed that all the mutations present in the S2 domain of Omicron BA.1 S decreased S protein expression on the cell surface, except for N764K polymorphism, when incorporated in the WA1^614G^ S protein. Interestingly, mutation N856K had the highest impact on the reduction of both cell–cell fusion and surface expression of S. To validate these findings, we next generated an Omicron BA.1 S protein containing the ancestral amino acid present in WA1 S protein at position 856 (S:K856N). Backmutation of S:856K to 856N resulted in a significant increase in fusogenicity and S cell surface expression as compared to Omicron BA.1 wild type S protein (Figure 4(F–G) and Supplementary Table 1). Taken together, these results demonstrated that mutations located in the S2 domain of Omicron BA.1 variant, in particular the S:N856K, reduced the expression of Omicron S on the cell surface and confirmed a strong relationship between the levels of S protein on the cell surface and the ability to induce cell–cell fusion.

### First-generation

Omicron subvariants have progressively evolved to an enhanced cell–cell fusion and increased S protein expression on the surface of infected cells.

**Figure 5.**
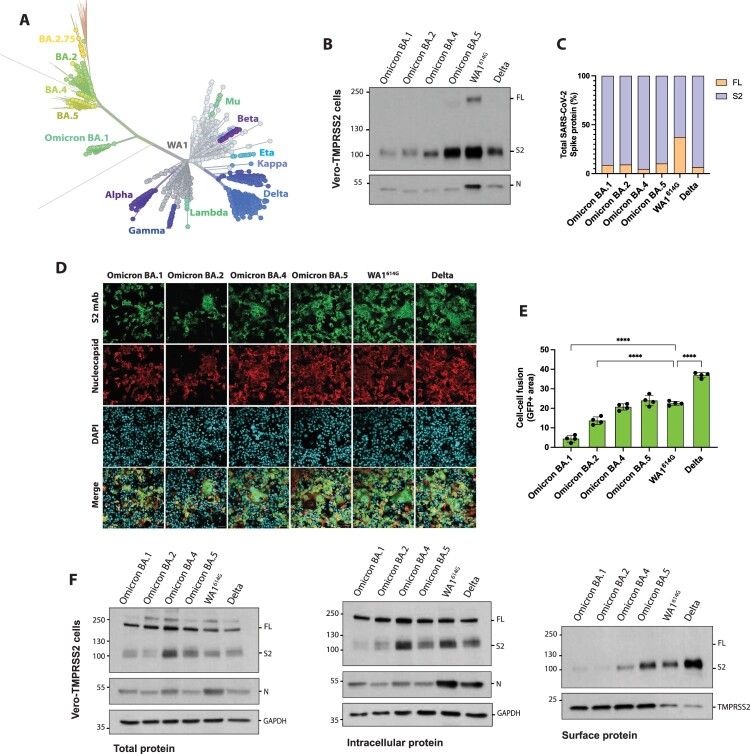
*In vitro* characterization of “first-generation” Omicron subvariants. (A) Phylogenetic tree of Omicron subvariants and other SARS-CoV-2 circulating variants. Unrooted tree was generated using Nextstrain and analysis was performed using 2640 genomes (GISAID database) sampled worldwide between December 2019 and August 2022. (B) Western Blotting of S protein cleavage of the different Omicron subvariants. Delta and ancestral WA1^614G^ variant were included as controls. Infections were performed in Vero-TMPRSS2 cells at an MOI of 0.01 and supernatants were collected at 48 h.p.i. Full-length (FL) spike protein (180 kDa), S2 cleaved spike (95 kDa), and nucleocapsid (N; 50 kDa) were detected using specific antibodies. Levels of N protein were used as loading control. (C) Quantification of full-length and S2 cleaved spike protein. Spike protein levels were normalized to nucleocapsid expression. (D) Syncytia formation induced by different Omicron subvariants, Delta and WA1^614G^ variants. Vero-TMPRSS2 cells were infected at an MOI of 0.01 and after 24 h.p.i, cells were fixed, and immunofluorescence assay was performed. Spike protein was detected using a S2 monoclonal antibody (green), N protein was detected using a polyclonal antiserum (red), and DAPI was used to stain the nucleus. Images were obtained using confocal microscopy. Scale bars, 50 μm. (E) Quantification of syncytia formation by each S variant represented as GFP positive area. Shown are the means and SDs of four replicates. One-way ANOVA with Dunnett’s multiple comparison test was performed to compare mean differences between each S variant and WA1^614G^ S. Statistical significance was considered when *p* ≤ 0.05 (*p < 0.05, **p < 0.01, ***p < 0.001, ****p < 0.0001). (F) Cleavage efficiency and S protein abundance of Omicron subvariants analysed by Western Blot. Vero-TMPRSS2 cells were infected at an MOI of 1 and biotinylation assay was performed after 24 h.p.i. Total, intracellular and surface protein fractions were collected. Full-length (FL) spike protein (180 kDa), S2 cleaved spike (95 kDa), nucleocapsid (N; 50 kDa), GADPH (37 kDa) and TMPRSS2 (21 kDa) were detected using specific antibodies. Levels of N, GAPDH and TMPRSS2 protein were used as loading control.

The Omicron BA.1 variant emerged at the end of November 2021 in South Africa [1]. Since then, “first-generation” Omicron sublineages have emerged (Figure 5(A)). The Omicron BA.2 subvariant was first detected in January 2022 and rapidly became dominant in several countries. Subsequently, the Omicron BA.4 and BA.5 subvariants were detected in South Africa in February 2022 and rapidly spread worldwide. This evolution drove the selection of mutations in both the S1 and S2 domains, and BA.2, BA.4 and BA.5 harbour only 4 out of the 6 mutations located in the S2 domain of Omicron BA.1 S protein: N764K, D796Y, Q954H and N969K, while the N856K and L981F polymorphisms were reverted to ancestor Wuhan-like variants. To investigate whether these amino acid reversion to ancestor variants could have an impact on S processing, we next characterized the “first-generation” Omicron subvariants. For this, we first investigated their S protein cleavage using supernatants from Vero-TMPRSS2 infected cells. As shown in Figure 5(B–C), all Omicron subvariants showed efficient cleavage similar to that of Delta S. However, there were clear differences and the total levels of the cleaved S2 form increased in supernatants from infected cells accordingly to the emergence and evolution of Omicron subvariants in humans, with a gradient of S2 total expression levels in supernatants from infected cells in this order: BA.1 > BA.2 > BA.4 > BA.5. Next, we assessed the syncytia formation induced by these subvariants using immunofluorescence microscopy of infected Vero-TMPRSS2 cells (Figure 5(D)) and then quantified syncytia using the cell–cell fusion assay (Figure 5(E)). Results showed that the fusogenic activity of Omicron subvariants progressively increased, with Omicron BA.1 showing the lowest cell–cell fusion and Omicron BA.5 exhibiting the highest S fusogenicity. To note, comparable levels of syncytia formation for Omicron BA.5 and WA1^614G^ control were found.

Finally, we assessed the levels of S protein expression on the surface of infected cells by using a protein biotinylation assay. For this purpose, Vero-TMPRSS2 cells were infected at an MOI of 1 with the different Omicron subvariants and controls. After 24 h.p.i, cell surface proteins were labelled with biotin and isolated using avidin agarose beads. Next, total protein extracts, intracellular and surface protein fractions were subjected to Western Blot analysis and probed against S protein (Figure 5(F)). We observed no significant differences in total S protein levels in infected cells of Omicron subvariant compared to WA1^614G^ and Delta controls. Similarly, intracellular S protein levels were comparable among the different variants with slightly increase in the S2 amount for Omicron BA.4. Interestingly, the levels Omicron BA.1 and BA.2 S protein expressed on the surface of infected cells were significantly lower compared to the rest. In contrast, we observed higher amounts of S2 protein expressed on the surface of Omicron BA.4 and BA.5 infected cells. Consistent with our cell–cell fusion experiments, Omicron BA.5 showed comparable levels of total S2 surface protein compared to WA1^614G^. Moreover, we found that Delta variant, which induces high levels of syncytia formation, had the highest S2 expression on the cell membrane.

### Evolution of “second generation” Omicron subvariants led to a diversification of lineage-specific fusogenic phenotype

**Figure 6.**
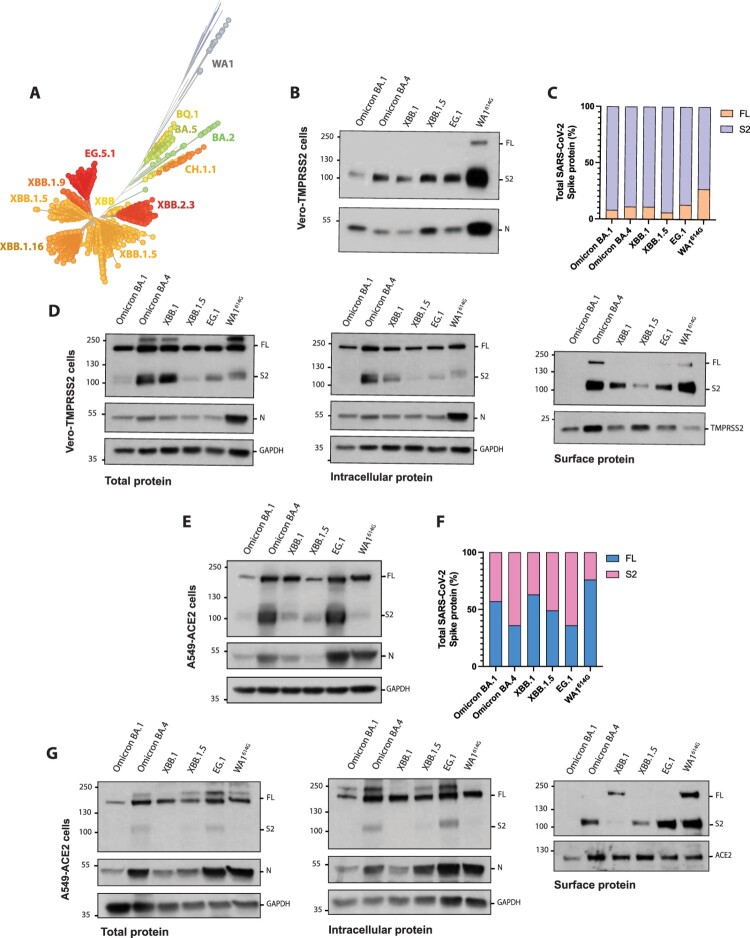
*In vitro* characterization of “second-generation” Omicron subvariants. (A) Phylogenetic tree of emerging BQ, XBB and EG Omicron sublineages. Unrooted tree was generated using Nextstrain and analysis was performed using 3041 genomes (GISAID database) sampled worldwide between September 2022 and November 2023. (B) Western Blotting of S protein cleavage of XBB and EG sublineages. Omicron BA.1, BA.4, and ancestral WA1^614G^ variants were included as controls. Infections were performed in Vero-TMPRSS2 cells at an MOI of 0.01 and supernatants were collected at 48 h p.i. Full-length (FL) spike protein (180 kDa), S2 cleaved spike (95 kDa), and nucleocapsid (N; 50 kDa) were detected using specific antibodies. Levels of N protein were used as loading control. (C) Quantification of full-length and S2 cleaved spike protein. Spike protein levels were normalized to nucleocapsid expression. (D) Cleavage efficiency and S protein abundance of XBB and EG Omicron sublineages analysed by Western Blot. Vero-TMPRSS2 cells were infected at an MOI of 1 and biotinylation assay was performed after 24 h.p.i. Total, intracellular and surface protein fractions were collected. Full-length (FL) spike protein (180 kDa), S2 cleaved spike (95 kDa), nucleocapsid (N; 50 kDa), GADPH (37 kDa) and TMPRSS2 (21 kDa) were detected using specific antibodies. Levels of N, GAPDH and TMPRSS2 protein were used as loading control. (E) Western Blotting of S protein cleavage of XBB and EG sublineages. Infections were performed in A549-ACE2 cells at an MOI of 0.1 and cell extracts were collected at 72 h.p.i. Full-length (FL) spike protein (180 kDa), S2 cleaved spike (95 kDa), and nucleocapsid (N; 50 kDa) were detected using specific antibodies. Levels of N protein were used as loading control. (F) Quantification of full-length and S2 cleaved spike protein in A549-ACE2 cells. Spike protein levels were normalized to nucleocapsid expression. (G) Cleavage efficiency and S protein abundance of XBB and EG Omicron sublineages analysed by Western Blot. A549-ACE2 cells were infected at an MOI of 1 and biotinylation assay was performed after 48 h.p.i. Total, intracellular and surface protein fractions were collected. Full-length (FL) spike protein (180 kDa), S2 cleaved spike (95 kDa), nucleocapsid (N; 50 kDa), GADPH (37 kDa) and ACE2 (92 kDa) were detected using specific antibodies. Levels of N, GAPDH and ACE2 protein were used as loading control.

Since the emergence of Omicron BA.5, more transmissible “second generation” Omicron subvariants have been arising (Figure 6(A)). These subvariants diversified from Omicron BA.2 sublineage and are characterized by increased transmissibility rates and immune escape potential [29,41]. While Omicron BQ.1 variant convergently evolved from BA.5, XBB sublineages were originated through recombination of two BA.2 descendants. More recently, EG subvariant diverged from XBB strains and rapidly spread worldwide. This high evolution rate was mainly reflected in the accumulation of antigenic mutations in the S1 domain, while the S2 subunit has remained more stable, and only some additional polymorphisms have been reported. Similar to “first-generation” BA.2, BA.4 and BA.5 Omicron sublineages, all “second generation” variants harbour N764K, D796Y, Q954H and N969K polymorphisms in the S2 domain. Additionally, the I1210T and T883I polymorphisms can also be found in the S2 domain of XBB.1 and XBB1.5 sublineages, respectively (Supplementary Figure 3).

In the context of this rapid evolving epidemiological situation, we next characterized the S protein cleavage, syncytia formation and S cell surface expression of a subset of “second generation” Omicron subvariants. For this, we first performed infections in Vero-TMPRSS2 cells with XBB.1, XBB.1.5 and EG.1 subvariants. Additionally, we included Omicron BA.1 as poorly fusogenic variant with low S cell surface expression; and Omicron BA.4 and WA1^614G^ as highly fusogenic controls with enhanced levels of S expressed on the surface of infected cells. As shown in Figure 6(B,C) and Supplementary Figure 1C, XBB.1, XBB.1.5 and EG.1 subvariants were efficiently cleaved as compared to controls. Next, biotinylation assays were performed to investigate the levels of S protein expressed by these Omicron subvariants on the surface of infected cells (Figure 6(D)). We found that levels of full-length S protein were similar among all SARS-CoV-2 variants tested, with an increased in S2 cleaved form of the S in the case of Omicron BA.4 and XBB.1 strains. Similarly, intracellular S2 cleaved S levels were higher in Omicron BA.4 and XBB.1 subvariants. Consistent with our previous findings, Omicron BA.1 showed minimum levels of S expressed on the surface of the Vero-TMPRSS2 infected cells, while higher surface amounts of S2 were observed in Omicron BA.4 and WA1^614G^ infected cells. Interestingly, XBB.1 and EG.1 showed higher expression levels of S2 cleaved S protein on the cell surface as compared to Omicron BA.1, but to a lesser extent as compared to BA.4 and WA1^614G^ controls. Additionally, we found that the XBB.1.5 subvariant had the lowest levels of S cell surface expression among all “second generation” subvariants tested. Remarkably, the ability of these variants to induce syncytia formation was in apparent accordance with the levels of S expressed on the surface of infected cells (Supplementary Figure 4).

Finally, and to validate our results, we performed similar infections experiments in A549-ACE2 cells, a human airway epithelial cell line. Consistently, we found that all SARS-CoV-2 variants were efficiently processed since S2 cleaved S protein was detected for all variants in the cell extracts from A549-ACE2 infected cells (Figure 6(E,F)). Next, protein biotinylation assays were performed after 72 h.p.i. (Figure 6(G)). Interestingly, only S2 cleaved form of the S protein was observed for Omicron BA.4 and EG.1 in both total and intracellular fractions. Nonetheless, we detected that these SARS-CoV-2 variants had similar S cell surface expression profile in A549-ACE2 cells as the one observed in Vero-TMPRSS2 infected cells; with the exception of XBB.1, where only full-length S protein was detected, suggesting perhaps a higher dependance of this subvariant on TMPRSS2 protease.

## Discussion

We have previously shown that polymorphisms in the S protein of SARS-CoV-2 viruses can influence S cleavage efficiency and syncytia formation [22]. However, the functional consequences of these amino acid changes on viral transmissibility and evolution are not clear. In this study, we expand on our previous results and investigate the S protein cleavage and fusogenicity of “first generation” and “second generation” Omicron sublineages. We found that S mutations 655Y, 679K, 681H and A701V located around the furin cleavage site of Omicron S protein enhance the fusogenic activity when introduced in WA1^614G^ S. Surprisingly, infection experiments showed that Omicron BA.1 subvariant was poorly fusogenic and induced low levels of syncytia formation despite containing some of these mutations. Using chimeric S proteins with different combinations of the S1 and S2 domains from WA1, Delta and Omicron BA.1 variants, we identified the S2 domain as responsible for this phenotype (Figure 4(A–C)). Some preliminary reports found that the N969K substitution contained in the S2 domain of Omicron BA.1 could be responsible [30], however we found that additional mutations like the S:N856K could also play a role.

Our results indicated that the S2 domain of Omicron BA.1 subvariant decreases cell–cell fusion. This domain harbours six unique polymorphisms not detected before in other previous circulating VOCs. While we showed that this domain was associated with low fusogenicity, we also found a direct link to lower S protein cell surface expression (Figure 4(D–E)), which likely explains the reduced cell-to-cell fusogenic activity. Omicron BA.1 S protein which harbours the N856K polymorphism, was less expressed on the cell membrane of infected cells as compared to other “first generation” and “second generation” Omicron subvariants and previous circulating WA1^614G^ and Delta strains (Figure 5(F) and Figure 6(D and G)), suggesting perhaps an intracellular retention of Omicron BA.1 S on the infected cell. It has been shown that the cytoplasmic tail of the S protein plays a role in intracellular trafficking and contains different binding sites for host trafficking proteins such as COP vesicles [19,42]. It is possible that the acquisition of mutations in the S2 domain such as N856K and N969K, could impact the recognition of the S protein by host factors that mediate the protein transport and therefore preventing the translocation of the S to the plasma membrane of the infected cells. Interestingly, the “first generation” Omicron BA.2, BA.4 and BA.5 subvariants induced higher levels of syncytia formation as compared to BA.1. The cell–cell fusion progressively increased among subvariants according to the timeline of emergence in the human population (Figure 5(D–E)) and in line with an increase of total levels of S protein on the surface of the infected cells (Figure 5(F)). Moreover, all “second generation” Omicron subvariants showed a significantly higher S expression on the cell surface as compared to Omicron BA.1 (Figure 6 and Supplementary Figure 4), although some differences were detected among subvariants. Interestingly, XBB.1, a variant resulted from recombination between BA.2.75 and BJ.1 sublineages showed an impaired ability to get cleaved in A549-ACE2 but not in Vero-TMPRSS2 cells. This potential dependence on TMPRSS2 protease, perhaps as a result of the recombination process, was overcome in the subsequent XBB.1.5 and EG.1 lineages that evolved to higher S2 expression on cell surface and syncytia formation. Importantly, BA.2, BA.4 and BA.5 and all “second generation” Omicron sublineages don’t contain the N856K mutation in their S2 domain, which could partially explain an increased ability to induce cell–cell fusion. Additionally, some of these differences could be attributed to the presence of mutations outside the S glycoprotein. It has been described that the non-structural protein 6 (nsp6), a viral factor involved in the biogenesis of double-membrane vesicles, and the S protein work in concert to drive viral pathogenicity and fitness [43–45]; and only SARS-CoV-2 chimeric viruses harbouring the S and nsp6 proteins from Omicron BA.1 fully recapitulate the infectivity and *in vivo* pathogenesis of Omicron BA.1 wild type virus. Moreover, recent reports have also evaluated the potential role of polymorphism in ORF9b, a viral protein that impairs host mitochondrial function to evade immune recognition, in the pathogenicity of XBB and EG sublineages [46]. Whether the appearance of compensatory mutations in non-S protein could determine SARS-CoV-2 evolution remains unknown.

Syncytia formation has been extensively associated with an enhanced pathology and lung damage in COVID-19 patients [17,18,20]. This cell–cell fusion with neighbouring cells relies on the efficient S protein cleavage and expression on the cell surface. While some studies have suggested that Omicron variant was not efficiently cleaved [47-49], we demonstrated otherwise, as all Omicron S protein tested showed optimal cellular processing (Figures 3, 5 and 6(B–C)). However, we found differences in the amount of S2 cleaved S in supernatants. This could suggest different levels of primed S protein expressed on the surface of the released viral particles, which could contribute to the higher infectivity and ACE2 binding observed in Omicron variants. Since we demonstrated competent cleavage, increased fusogenicity and S surface expression on infected cells as compared to Omicron BA.1, we could hypothesize that Omicron sublineages are progressively becoming more pathogenic over time. However, contradictory studies can be found in the field and detailed clinical data from patients infected with the latest Omicron variants is missing. Some *in vivo* studies using clinical isolates [50] and chimeric recombinant viruses harbouring Omicron BA.4/5 S protein [51] have suggested that BA.4 and BA.5 are more pathogenic and induced higher levels of inflammation in the hamster model compared to previous Omicron sublineages. In contrast, Uraki *et al.* [52] found that BA.4 and BA.5 subvariants have similar pathogenicity to BA.2 in rodents. Similarly, recent studies have shown that the pathogenicity of XBB.1 in hamsters is comparable to previous circulating BA.2 sublineages [29]; and that the recently emergence EG.5.1 variant exhibits comparable fusogenicity and pathogenicity in hamsters as XBB.1 and XBB.1.5 subvariants [46]. It will be important to investigate whether this increase in fusogenic activity could reflect in an enhancement on Omicron pathogenesis in humans.

The role of S cell-surface expression, if any, in promoting virus replication *in vivo* is unclear. SARS-CoV-2 particle assembly and budding have been shown to take place in the ER-Golgi, with virions egressing via lysosomal trafficking [53]. Thus, virus particles acquire their membrane and therefore their S proteins at the ER-Golgi compartment, and not at the plasma membrane. Nevertheless, cell-to-cell fusion due to S surface expression facilitates spreading of infection in tissues in the absence of virus particle formation. This virion-independent form of spreading might be less sensitive to antibody neutralization. Moreover, infection of cells by fusion to an infected cell results in the direct delivery in the newly infected cell of non-structural viral proteins previously expressed in the first infected cell, which might facilitate their host modulatory functions. In any case, more research is required to better understand the advantages of cell-to-cell fusion for viruses.

Our results could also have implications on the improvement and development of COVID-19 vaccines. Current mRNA COVID-19 vaccines contained the mRNA encoding for the S protein of SARS-CoV-2 as a main antigen. These vaccines contained only Wuhan-like S mRNA at the beginning of the pandemic. However, they have been recently updated to match circulating variants in humans and currently bivalent formulation containing both the Wuhan-like and one of the most recent Omicron S variants are used. After vaccine administration, the Omicron S protein-encoding mRNA is delivered via lipid nanoparticles (LNPs) to human cells. After internalization of the LNP, the mRNA is released in the cytoplasm and translated by the ribosomes attached to the ER to produce mature S proteins. The S protein is then trafficked to finally express on the cell membrane, where it gets exposed and recognized by the human immune system. This will trigger the production of anti-S antibodies and prime for specific cellular immunity. We found that mutations in the S2 domain of Omicron S protein decreased the S expression on the surface of infected cells. In particular, the N856 K mutation found in Omicron BA.1 subvariant could negatively impact the expression of the S protein on the cell surface. Additionally, polymorphisms such as the N969 K and Q954H present in all Omicron sublineages including Omicron BA.4/BA.5 and the recently emerged XBB and EG could also play a role. The presence of these S2 mutations could reduce the effectiveness of current and future booster COVID-19 vaccines. A potential way to overcome this, would be the formulation of bivalent vaccines containing chimeric S proteins that harbour the S1 domain of the most recent circulating Omicron variant and the S2 domain of a Wuhan-like strain. This would allow a higher S expression on the cell surface and therefore, an increased antibody production and enhanced cellular immune responses. Further research is needed to understand and explore the role of S2 mutations on COVID-19 vaccines design.

## Supplementary Material

SupFigures_R1

SupTable1_R1

## Data and materials availability

All data are available in the main text or the supplementary materials. Further information and requests for resources and reagents should be directed to and will be fulfilled by the lead contact, Adolfo García-Sastre (adolfo.garcia-sastre@mssm.edu).
