# Supplementary material for: The impact of S2 mutations on Omicron SARS-CoV-2 cell surface expression and fusogenicity
Source: Emerg Microbes Infect. 2023 Dec 19;13(1):2297553. doi: 10.1080/22221751.2023.2297553 (PMC10866063; doi:10.1080/22221751.2023.2297553)
Supplement: SupFigures_R1 [file TEMI_A_2297553_SM8978.pdf]

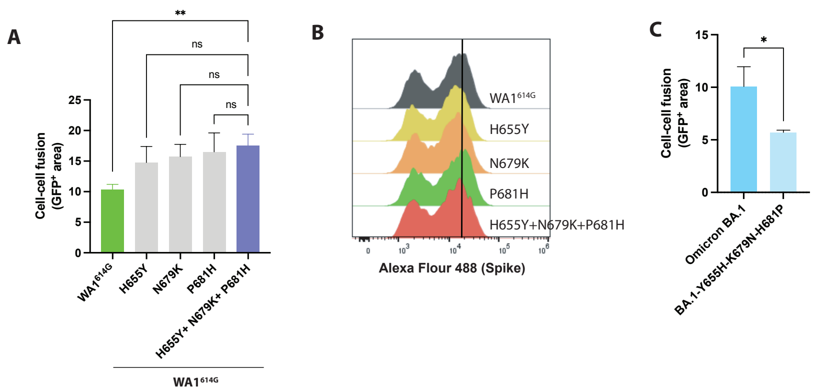

Supplementary Figure 1. Mutations around the furin cleavage site of Omicron BA.1 variant enhance cell-cell fusion but do not impact on S cell surface expression. A) Quantification of syncytia formation of WA1^{614G} wild type S, WA1^{614G} S containing mutation H655Y, N679K or P681H and WA1^{614G} S containing the combination of the three mutations. Cell-cell fusion was represented as GFP positive area. Shown are the means and SDs of four replicates. One-way ANOVA with Holm-Šídák multiple comparisons test was performed to compare mean differences between each S variant and the triple WA1^{614G} - H655Y, N679K or P681H S mutant. Statistical significance was considered when $p \leq 0.05$ (*$p < 0.05$, **$p < 0.01$, ***$p < 0.001$, ****$p < 0.0001$). **B)** Spike surface expression of S proteins analyzed by flow cytometry. HEK293T cells were transfected with the corresponding S proteins and after 24 hours, cells were stained with anti-S antibody. **C)** Quantification of syncytia formation of Omicron BA.1 wild type S protein and a mutant Omicron BA.1 S protein harboring the ancestral amino acids at position 655, 679 and 681 (BA.1-Y655H-K679N-H681P). Cell-cell fusion was represented as GFP positive area. Shown are the means and SDs of four replicates. Unpaired two-tailed t test was performed to compare mean differences between wild type and mutant Omicron BA.1 S proteins (*$p < 0.05$).

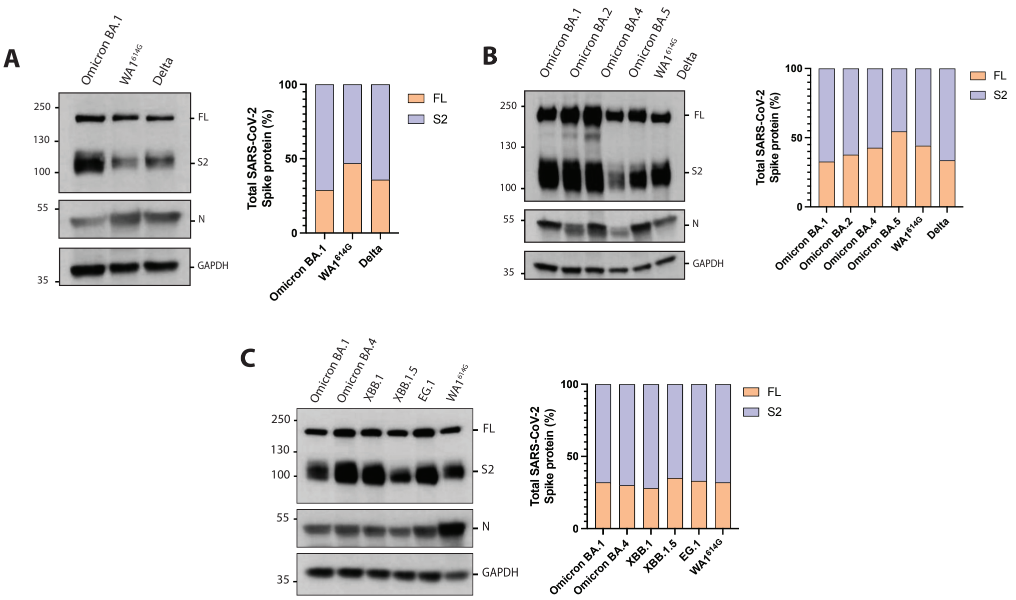

Supplementary Figure 2. Spike cleavage efficiency of Omicron subvariants in cell extracts from Vero-TMPRSS2 infected cells. Western Blotting of S protein cleavage of ‘first-generation’ Omicron subvariants **(A-B)** and ‘second-generation’ Omicron subvariants **(C)**. Full-length (FL) spike protein (180 kDa), S2 cleaved spike (95 kDa), nucleocapsid (N; 50 kDa) and GAPDH (37 kDa) were detected using specific antibodies. Levels of N and GAPDH protein were used as loading control. Quantification of full-length and S2 cleaved spike protein is also shown for each Western Blot.

A

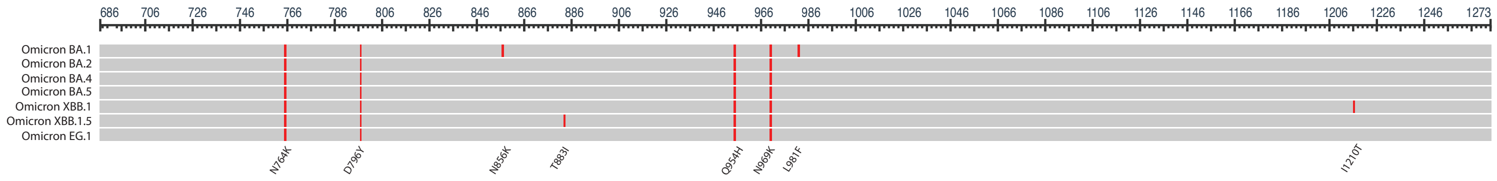

Supplementary Figure 3. Polymorphisms in the S2 domain of the 'first-generation' Omicron and 'second-generation' Omicron subvariants used in this study. A) Multiple sequence alignment of the S2 domains was performed using COBALT multiple alignment tool. Wuhan-1 S2 domain was used as a reference.

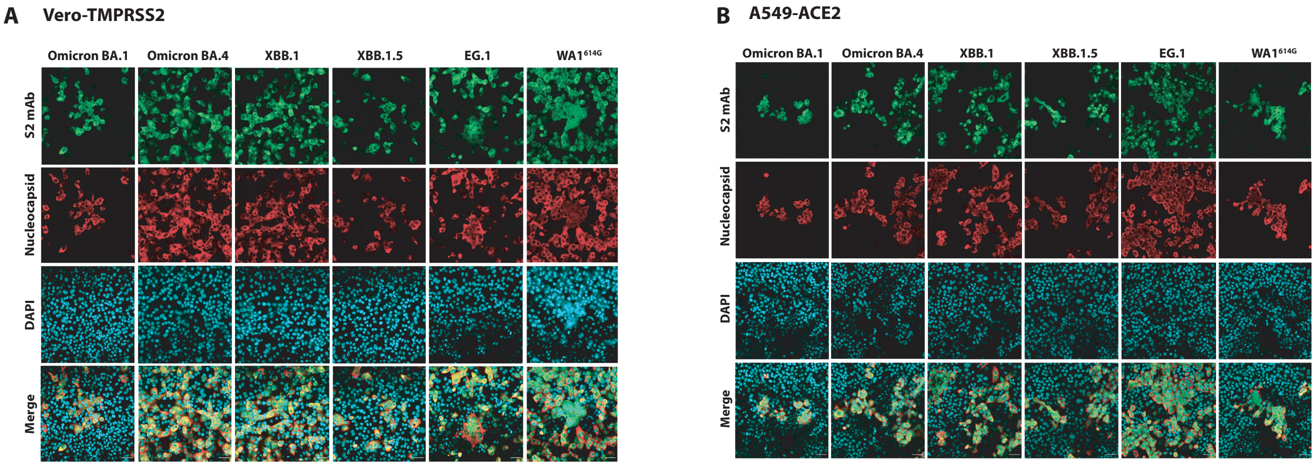

Supplementary Figure 4. Syncytia formation induced by 'second-generation' Omicron subvariants in Vero-TMPRSS2 (A) and A549-ACE2 cells (B). Vero-TMPRSS2 cells were infected at an MOI of 0.01 and after 24 h.p.i, cells were fixed, and immunofluorescence assay was performed. Alternatively, A549-ACE2 cells were infected at an MOI of 0.1 and fixed after 48 h.p.i. Spike protein was detected using a S2 monoclonal antibody (green), N protein was detected using a polyclonal antiserum (red), and DAPI was used to stain the nucleus. Images were obtained using confocal microscopy. Scale bars, 50 μm.
