# Supplementary material for: The impact of S2 mutations on Omicron SARS-CoV-2 cell surface expression and fusogenicity
Source: Emerg Microbes Infect. 2023 Dec 19;13(1):2297553. doi: 10.1080/22221751.2023.2297553 (PMC10866063; doi:10.1080/22221751.2023.2297553)
Supplement: SupTable1_R1 [file TEMI_A_2297553_SM8968.docx]

**Supplementary Table 1.** **Quantification of the levels of S protein expressed on the cell surface of HEK293T cells upon transfection with the S plasmids used in this study.** After 24 hours post-transfection, cells were stained with an anti-S Alexa Flour 488 antibody and fluorescence was captured by FACS. Then, Median Fluorescence Intensities (MFI) were calculated as a robust estimation of the signal intensity of the peak of each S plasmid.

| **S plasmids** | **MFI** |
| --- | --- |
| ***Figure 4C*** | |
| **WA1** | 14606 |
| **WA1/Delta** | 10492 |
| **WA1/Omicron** | 6412 |
| **Delta** | 19395 |
| **Delta/WA1** | 30602 |
| **Delta/Omicron** | 9525 |
| **Omicron** | 9236 |
| **Omicron/WA1** | 17553 |
| **Omicron/Delta** | 22963 |
| ***Figure 4E*** | |
| **WA1^614G^** | 14606 |
| **Omicron BA.1** | 9236 |
| **N764K** | 16904 |
| **D796Y** | 10772 |
| **N856K** | 8350 |
| **Q954H** | 11738 |
| **N969K** | 11232 |
| **L981F** | 12026 |
| **N856K + Q954H + N969K** | 6179 |
| ***Figure 4G*** | |
| **Omicron BA.1** | 7240 |
| **WA1^614G^** | 10757 |
| **BA.1-K856N** | 15317 |
